# Cloning and Characterization of EF-Tu and EF-Ts from *Pseudomonas aeruginosa*

**DOI:** 10.1155/2013/585748

**Published:** 2013-08-05

**Authors:** Stephanie O. Palmer, Edna Y. Rangel, Alberto E. Montalvo, Alexis T. Tran, Kate C. Ferguson, James M. Bullard

**Affiliations:** Chemistry Department, SCIE. 3.320, The University of Texas-Pan American, 1201 W. University Drive, Edinburg, TX 78541, USA

## Abstract

We have cloned genes encoding elongation factors EF-Tu and EF-Ts from *Pseudomonas aeruginosa* and expressed and purified the proteins to greater than 95% homogeneity. Sequence analysis indicated that *P. aeruginosa* EF-Tu and EF-Ts are 84% and 55% identical to *E. coli* counterparts, respectively. *P. aeruginosa* EF-Tu was active when assayed in GDP exchange assays. Kinetic parameters for the interaction of EF-Tu with GDP in the absence of EF-Ts were observed to be *K*_*M*_ = 33 **μ**M, *k*_cat_^obs^ = 0.003 s^−1^, and the specificity constant *k*_cat_^obs^/*K*_*M*_ was 0.1 × 10^−3^ s^−1^ **μ**M^−1^. In the presence of EF-Ts, these values were shifted to *K*_*M*_ = 2 **μ**M, *k*_cat_^obs^ = 0.005 s^−1^, and the specificity constant *k*_cat_^obs^/*K*_*M*_ was 2.5 × 10^−3^ s^−1^ **μ**M^−1^. The equilibrium dissociation constants governing the binding of EF-Tu to GDP (*K*_GDP_) were 30–75 nM and to GTP (*K*_GTP_) were 125–200 nM. EF-Ts stimulated the exchange of GDP by EF-Tu 10-fold. *P. aeruginosa* EF-Tu was active in forming a ternary complex with GTP and aminoacylated tRNA and was functional in poly(U)-dependent binding of Phe-tRNA^Phe^ at the A-site of *P. aeruginosa* ribosomes. *P. aeruginosa* EF-Tu was active in poly(U)-programmed polyphenylalanine protein synthesis system composed of all *P. aeruginosa* components.

## 1. Introduction

*Pseudomonas aeruginosa* is an opportunistic bacterial pathogen and the causative agent in a wide range of infections, including bacteremia, urinary tract infections, burn wound infections, and pulmonary infections in patients on respirators. In the intensive care unit, *P. aeruginosa* is the most common Gram-negative pathogen causing infections, accounting for 10% of all cases. In hospitals, *P. aeruginosa* is responsible for about one-seventh of all infections, with multidrug-resistant strains becoming increasingly common  [1, 2]. However, the most serious medical problem caused by *P. aeruginosa* is lung infection associated with cystic fibrosis [3]. The lungs of cystic fibrosis patients are commonly colonized by *P. aeruginosa* before ten years of age, and chronic infections are the most important causes of morbidity and mortality [4]. Antimicrobial resistance among clinical isolates of *P. aeruginosa* is significant and growing [5] and has become a major problem in hospital patients [6].

Protein synthesis is an essential metabolic process occurring in all bacteria and is a validated target for the development of new antibiotics [7, 8]. The components of the bacterial protein synthesis system are sufficiently different at the molecular level from those found in eukaryotic cells as to allow development of compounds that inhibit growth of bacteria without having adverse effects on eukaryotic cells. Elongation factor Tu (EF-Tu) plays a central role in protein biosynthesis by delivering aminoacyl-tRNA (aa-tRNA) to the A-site of the ribosome during the elongation phase of protein synthesis [9]. To this end, EF-Tu forms a ternary complex with GTP and aa-tRNA. The ternary complex binds the A-site of an actively translating ribosome in a mRNA-dependent manner. Once the cognate ternary complex is bound to the ribosome, the GTP is hydrolyzed to GDP by activation of the GTPase activity of EF-Tu  [10]. The resulting EF-Tu·GDP complex then dissociates from the ribosome and is recycled to the active EF-Tu·GTP complex in a nucleotide exchange reaction catalyzed by elongation factor Ts (EF-Ts)  [11].

 We have previously developed a poly(U)-directed aminoacylation/translation (A/T) high throughput screening system from *E. coli* which was used to identify a series of compounds containing a common scaffold (tetrahydropyrido[4,3-*d*]pyrimidin-4-ol) that inhibited protein synthesis in a broad spectrum of bacteria [12]. Since *P. aeruginosa *is arguably the organism most evolutionarily distant from *E. coli* among the major human Gram-negative pathogens, and given its importance as the causative agent of fatalities among cystic fibrosis patients, we are developing an A/T high throughput screening system similar to the *E. coli* system but consisting solely of components from *P. aeruginosa*. Also, several compounds have recently been identified that inhibit the activity of EF-Tu in Gram-positive organisms  [13, 14]. These findings provided an impetus for us to better understand the components involved in protein synthesis as it occurs in *P. aeruginosa* and provided evidence that other components besides the ribosome in the protein synthesis apparatus can be important targets in the development of antibacterials. We describe here the cloning and enzymatic characterization of EF-Tu and EF-Ts from *P. aeruginosa* as part of the continuing development of a *P. aeruginosa* A/T screening system.

## 2. Methods and Materials

### 2.1. Materials

Champion pET Directional TOPO Expression Kits were from Invitrogen. Plasmids were from Novagen. Oligonucleotides were from Integrated DNA Technologies (Coralville, IA, USA). All other chemicals were obtained from either Sigma Aldrich (St. Louis, MO-) or Fisher Scientific (Pittsburg, PA-). Early log-phase ribosomes from *P. aeruginosa* strain PA01 were prepared in the laboratory of Walter Hill at the University of Montana (Missoula, MT-) as previously described [15]. DNA sequencing was at the Howard Hughes Medical Institute (HHMI) laboratory at the University of Texas-Pan American.

### 2.2. Gel Electrophoresis and Protein Analysis

Sodium dodecyl sulfate-polyacrylamide gel electrophoresis (SDS-PAGE) was performed using 4% to 12% (w/v) polyacrylamide precast gels (Bio-Rad). Benchmark unstained protein molecular weight markers were from Invitrogen (Madison, WI,USA). Protein concentrations were determined by the method of Bradford  [16] using Coomassie Protein Assay Reagent (Thermo Scientific) and bovine serum albumin as the standard.

### 2.3. Cloning and Purification of EF-Tu and EF-Ts

The gene encoding EF-Tu was amplified by PCR (Bio-Rad MJ Mini Therma Cycler) from genomic DNA isolated in our laboratory from *P. aeruginosa* PAO1 (ATCC) using a forward primer (5′-CACCATGGCTAAAGAAAAATTTGAACG-3′) which contained the 5′ CACC sequence for insertion into pET101/D-TOPO directional plasmid and a reverse primer (5′-TCAATGGTGATGGTGATGGTGAGAACC TTCGATGATCTTGGCAACC-3′) which was designed to add six histidine amino acid residues to the C terminus of EF-Tu. The PCR product was inserted into pET101/D-TOPO and transformed into *E. coli* Rosetta 2(DE3) Singles Competent Cells (Novagen). The gene encoding EF-Ts was amplified from *P. aeruginosa* genomic DNA using a forward primer (5′-CTGAGCTAGCGCAGAAATTACTGCAGCCAT-3′) designed to add an *Nhe*I restriction site to the 5′ end of the gene and a reverse primer (5′-GACTAAGCTTCATTACTGCTTGGTGGCGG-3′) which was designed to add a *Hind*III restriction site to the 3′ end of the gene. This allowed the PCR product to be placed downstream on a region containing a sequence encoding six histidine amino acid residues. The PCR product was inserted between the *Nhe*I/*Hind*III restriction sites in pET-28b(+) (Novagen) and transformed into Rosetta 2(DE3) Singles Competent Cells. All primers were designed from *P. aeruginosa* complete genome sequence listed on the National Center for Biotechnology Information website (NC_002516.2).

Cultures were grown in F-medium  [17] containing 50 *μ*g/mL of ampicillin and 75 *μ*g/mL of chloramphenicol at 37°C. Expression of the target proteins was induced at an optical density (A_600_) of 0.6 by the addition of isopropyl *β*-D-1-thiogalactopyranoside (IPTG) to 0.5 mM. Growth of the bacterial culture was continued for 1.5 h after induction, and the bacteria were harvested by centrifugation (4000 ×g, 60 min, 4°C). The cells were lysed, and fraction I was prepared as previously described  [17]. *P. aeruginosa* EF-Tu precipitated with proteins between 45% and 60% saturation of ammonium sulfate, and the precipitated protein was collected by centrifugation (23,000 ×g, 60 min, 4°C). EF-Tu was further purified to more than 98% homogeneity using nickel-nitrilotriacetic acid (NTA) affinity chromatography (Perfect Pro, 5 Prime). The column (1 mL) was washed with buffer A (20 mM Hepes (pH 7.0), 40 mM KCl, 1 mM MgCl_2_, 0.1 mM EDTA, 10% Glycerol) containing 1 M NaCl, and 20 mM imidazole, and the target protein was eluted in buffer A containing 200 mM imidazole. Elution of the protein was followed by dialysis (two times) against buffer A. Purified proteins were fast frozen in liquid nitrogen and stored at −80°C. *P. aeruginosa* EF-Ts did not precipitate in ammonium sulfate concentrations below 60% saturation, and the supernatant from the 60% saturated ammonium sulfate sample was used for the further purification of EF-Ts using NTA affinity chromatography as described earlier for EF-Tu. EF-Ts was purified to greater than 95% homogeneity.

### 2.4. Determination of the Concentration of GDP in *P. aeruginosa* EF-Tu Preparations

The concentration of GDP in *P. aeruginosa* EF-Tu preparations was determined by absorbance spectroscopy. Samples of EF-Tu preparations (250 *μ*L, 37 *μ*M) and controls (dialysis buffer) were denatured at 90°C for 10 min. The denatured samples were centrifuged in an Eppendorf centrifuge for 30 min at 4°C to remove the precipitated protein. The absorbance spectra of the supernatants containing released GDP were then measured using the dialysis buffer control as the background. The concentrations of GDP were measured by the absorbance at 260 nm using an extinction coefficient of 13,804 M^−1^ cm^−1^  [18].

### 2.5. Aminoacylation of tRNA^Leu^ and tRNA^Phe^

tRNALeu was aminoacylated in 2.5 mL reactions containing 50 mM Tris-HCl, (pH 7.5), 5 mM MgOAc, 2.5 mM ATP, 90 *μ*M crude *E. coli* tRNA (approximately 7 *μ*M tRNALeu), 50 *μ*M [^3^H]leucine (60 cpm/pmol), and 0.8 *μ*M *P. aeruginosa* leucyl-tRNA synthetase (previously purified in our laboratory). tRNA^Phe^ was aminoacylated in 2.5 mL reactions containing 50 mM Tris-HCl, (pH 7.5), 5 mM MgCl, 2.5 mM ATP, 90 *μ*M crude *E. coli* tRNA (approximately 2 *μ*M tRNA^Phe^), 50 *μ*M [^3^H]phenylalanine (75 cpm/pmol), and 1.4 *μ*M *P. aeruginosa* phenyl-tRNA synthetase (previously purified in our laboratory). Both aminoacylation reactions were incubated at 37°C for 1.5 h. The tRNA was then collected by precipitation with ethanol followed by centrifugation (30,000 ×g, 45 min, 4°C) and resuspended in 0.5 mL of 10 mM Tris-HCl, (pH 7.5).

### 2.6. Determination of GDP Exchange by EF-Tu and of the Ability of EF-Ts to Stimulate This Exchange

Nitrocellulose filter binding assays were used to determine GDP exchange by EF-Tu as previously described  [19, 20]. Assays to determine the initial velocity of GDP exchange were in buffer B (50 mM Tris-HCl, (pH 7.5), 50 mM NH_4_Cl, and 10 mM MgCl_2_, 1.0 mM dithiothreitol (DTT)) containing 12 *μ*M [^3^H]GDP (500 cpm/pmol). Reactions were incubated at 37°C and were stopped by diluting the reaction mix to 2 mL in buffer B and immediately filtered through nitrocellulose (Whatman; Protran BA 85). Filters were washed three times with 2 mL of buffer B and allowed to dry, and the amount of EF-Tu·[^3^H]GDP complex retained was determined by scintillation counting (Beckman Coulter LS 6500). Initial velocity reactions were from 1 to 4 min, and GDP concentrations were varied as indicated. Initial velocity assays containing EF-Ts were assayed at reactions times between 15 and 75 s, and the concentration of EF-Ts was held constant at 0.01 *μ*M, while GDP concentrations were varied as indicated.

 EF-Ts stimulates the exchange of GDP bound by EF-Tu. Nitrocellulose binding assays used to determine EF-Ts stimulation of GDP exchange were the same as those described earlier with the following changes: GDP was reduced to 1 *μ*M, EF-Tu was held constant at 0.64 *μ*M, and EF-Ts was added to 0.05 *μ*M (1 : 13 EF-Ts to EF-Tu ratio), and the reaction times for the assays were decreased from 30 min to 15 to 120 s.

### 2.7. Equilibrium Dissociation Constants for the EF-Tu·GDP and EF-Tu·GTP Complexes

Reactions were carried out to determine the equilibrium dissociation constants (*K*_GDP_ and *K*_GTP_) for the interaction of *P. aeruginosa* EF-Tu with GDP and GTP. The reaction was carried out in buffer B containing 1.3 *μ*M total *P. aeruginosa* EF-Tu and 12 *μ*M [^3^H]GDP (500 cpm/pmol) or [^3^H]GTP (750 cpm/pmol). EF-Ts was added to 0.05 *μ*M as indicated. To form EF-Tu·nucleotide complexes, the reactions were incubated for 2 h at 0°C. To determine the total amount of complex formed (defined as active amounts of EF-Tu), reactions were immediately filtered through nitrocellulose filters following incubation. Reactions to determine *K*_GDP_ and *K*_GTP_ were diluted by the addition of 5 mL of buffer B that had been preincubated at 37°C, 23°C, or 0°C. Incubation was continued at those temperatures after dilution for 10, 20, and 30 min, respectively. Remaining amounts of the complexes were determined by filtration through nitrocellulose filters.

### 2.8. EF-Tu Ternary Complex Formation and Phe-tRNA^Phe^ Delivery to the Ribosome

Ternary complex formation was assayed by examining the ability of EF-Tu to protect [^3^H]Leu-tRNA^Leu^ ) from digestion by RNase A  [21, 22]. EF-Tu (0, 0.6, 0.9, 1.2, 1.5, 1.8, 2.1, and 2.4 *μ*M) was incubated in reaction mixtures (50 *μ*L) containing 1.2 *μ*M [^3^H]Leu-tRNA^Leu^, 0.5 mM GTP, 50 mM Tris-HCl, (pH 7.5), 1 mM DTT, 50 mM NH_4_Cl, and 6.5 mM MgCl_2_ for 15 min at 4°C. RNAase A was then added to a final concentration of 0.02 mg/mL and incubated for an additional 20 s. The reactions were stopped by the addition of 3 mL of ice-cold 5% trichloroacetic acid (TCA) and filtered through nitrocellulose filters (Whatman). Retention of [^3^H]Leu signified EF-Tu protection of [^3^H]Leu-tRNA^Leu^ from nuclease digestion  [22].

To determine ternary complex binding to the ribosome, a mixture containing 50 mM Tris-HCl, (pH 7.5), 0.1 mM spermine, 40 mM KCl, 4 mM MgCl_2_, 1.0 mM DTT, 0.3 mM GTP or guanosine 5′-[*β*,*γ*-imido]triphosphate (GDPNP), 0.15 mg/mL poly(U) mRNA, 0.06 *μ*M *P. aeruginosa *EF-Ts, 3.2 *μ*M EF-Tu, 1.0 *μ*M *P. aeruginosa* ribosomes, and 0.75 *μ*M [^3^H]Phe-tRNA^Phe^ (25 cpm/pmol) was used. The concentration of EF-Tu was varied from 0.32 to 1.6 *μ*M as indicated. All components were preincubated at 37°C for 15 min in the absence of ribosomes to allow ternary complex formation. Ribosomes were then added, and incubation was continued for an additional 15 min at the same temperature. The amounts of [^3^H]Phe-tRNA^Phe^ bound to the ribosome, representing ternary complex, were analyzed using glass microfiber filter (Whatman) binding as previously described  [23]. Control reactions lacked either EF-Tu or ribosomes.

### 2.9. EF-Tu Functions in Protein Synthesis

Protein synthesis assays were carried out in 50 *μ*L reactions containing 50 mM Tris-HCl, (pH 7.5), 10 mM MgCl_2_, 25 mM KCl, 4 mM phosphoenolpyruvate (PEP), 0.025 U/mL pyruvate kinase (PK), 1.5 mM ATP, 0.5 mM GTP, 40 *μ*M [^3^H]phenylalanine (75 cpm/pmol), 0.3 mg/mL poly(U) RNA, 0.03 mM spermine, 1 mM DTT, 0.05 *μ*M *P. aeruginosa* elongation factor-Ts (EF-Ts), 0.2 *μ*M *P. aeruginosa* elongation factor-G (EF-G), 0.1 *μ*M *P. aeruginosa* phenylalanyl-tRNA synthetase (PheRS), 0.2 *μ*M *P. aeruginosa* ribosomes, and the indicated amounts of *P. aeruginosa* EF-Tu. Reactions were started by the addition of *E. coli* tRNA to a final concentration of 0.5 *μ*M tRNAPhe and continued for 1 h at 37°C. Reactions were stopped by the addition of 2 mL 10% (w/v) TCA and filtered through glass fiber filters (Whatman) as previously described  [23]. Retention of [^3^H]Phe represents the amount of polyphenylalanine, poly (Phe), synthesized.

## 3. Results

### 3.1. Sequence Analysis

The translation elongation factors EF-Tu and EF-Ts previously studied have primarily been those from *E. coli* (and a few other bacteria) and from mammalian mitochondria and to a much lesser extent from the eukaryotic cytoplasm  [24, 25]. We analyzed the similarity between *P. aeruginosa* EF-Tu and EF-Ts and their homologs from other groups of organisms (Table 1). When compared with other Gram-negative bacteria (primarily from the phylum Proteobacteria), *P. aeruginosa* EF-Tu residues were observed to be highly conserved, with the percentage of identical residues ranging from 72% to 84%. However, the level of homology of *P. aeruginosa* EF-Ts with the same organisms ranged from only 31% to 55% identical residues. When compared with members of the Chlamydiales, Actinobacteria, and Gram-positive Firmicutes, the percent of identical residues did not differ significantly from that observed with the Gram-negative bacteria for both EF-Tu (67% to 73%) and EF-Ts (29% to 45%). However, when *P. aeruginosa* EF-Tu was compared to eukaryotic cytoplasmic EF-Tu (EF1*α*), the percent identity is only 26%, and when compared to the mitochondrial counterparts the homology was 37% identical residues. *P. aeruginosa* EF-Ts had negligible homology to eukaryotic EF-Ts (EF-1*β*). For the pursuit of compounds that specifically inhibit the activity of bacterial EF-Tu or EF-Ts, the lack of homology with eukaryotic homologs is an advantage.

**Figure 1:**
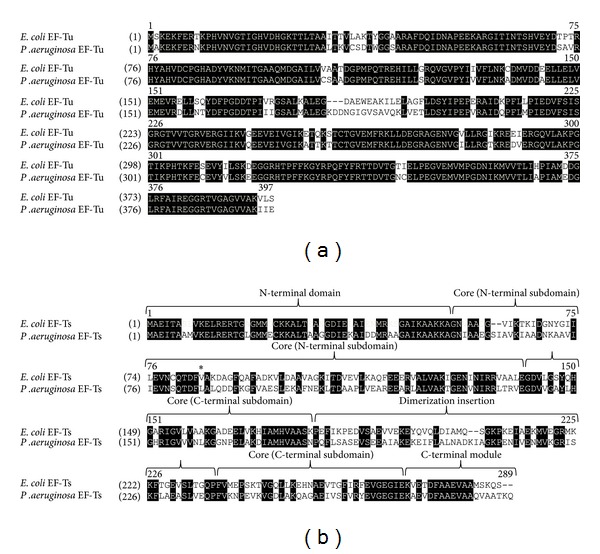
Alignment of *P. aeruginosa* EF-Tu and EF-Ts with *E. coli* homologs. (a) Alignment of *E. coli* and *P. aeruginosa* EF-Tu. Black background indicates conserved residues. (b) Alignment of *E. coli* and *P. aeruginosa* EF-Ts. The four structural modules are labeled. The asterisk indicates the residue of the strictly conserved TDFV sequence that contains a variant residue in all *Pseudomonas sp.* EF-Ts proteins.

The crystal structures of *E. coli* EF-Tu reveal that the protein folds into a three-dimensional structure composed of three domains connected by highly flexible spacer peptides  [26, 27]. The N-terminal 200 amino acids are encompassed within domain I which contains the guanine nucleotide binding site and the catalytic site for the GTPase activity. The functions of domain II (residues 208–295, *E. coli* numbering) and domain III (residues 300–393) are less well understood, but domains I and III interact with EF-Ts, while all three domains are involved in aa-tRNA binding [26, 28, 29]. Sequence comparisons show that EF-Tu from *P. aeruginosa* is 84% identical to EF-Tu from *E. coli* (Figure 1(a)). The primary region of amino acid sequence divergence appears to be within the region just N-terminal to the spacer connecting domains I and II. This region of sequence divergence is also observed in a larger alignment  [10] of EF-Tu molecules from Gram-negative organisms (data not shown).

 In contrast to the comparison for EF-Tu, the primary sequence of *P. aeruginosa* EF-Ts is only 55% identical to that of its *E. coli* homolog.  Figure 1(b) shows the high degree of divergence between *E. coli* and *P. aeruginosa* EF-Ts. However, the N-terminal and C-terminal modules of EF-Ts appear to contain more conserved residues than the other regions. These regions interact with nucleotide binding domain I of EF-Tu (which is highly conserved)  [26, 30]. Residues Asp-80, Phe-81, and Gly-126 and residues surrounding Ile-125 in subdomain N of the core region (amino acid residues that interact directly with residues in domain I of EF-Tu) are conserved between *E. coli* and *P. aeruginosa* EF-Ts. The core region contains a lesser degree of conserved residues. However, subdomain C located within the core region contains conserved residues His-147, Ile-151, Lys-166, Met-170, Ala-174, and Val-234 (*E. coli* numbering) that directly interact with amino acid residues in domain III of EF-Tu  [26]. Residues 180–228 (*E. coli* numbering) contain the smallest amount of conserved residues and are contained within a plausible dimerization module of EF-Ts  [26]. This region interacts with the same region of an identical EF-Ts in the proposed EF-Tu·EF-Ts dimer; thus, the low conservation of residues here is consistent with low homology between diverse EF-Ts molecules from various organisms. However, when this region is compared with the same regions from other members of the *Pseudomonas* genus, *P. mendocina*,* P. putida*,* P. fluorescens*,* and P. syringae*, it is highly conserved (data not shown). A unique feature was noted within the sequence of *P. aeruginosa* EF-Ts that involved the highly conserved TDFV motif which acts to displace the Mg^2+^ in the GTPase center of EF-Tu  [31]. In all organisms analyzed, this motif contains Thr-Asp-Phe-Val at positions 79–82 (*E. coli* EF-Ts numbering). However, in EF-Ts from all of the *Pseudomonas* strains listed earlier, the valine was replaced with a leucine. The addition of a methyl group to the side chain would seem to be a modest change, and in the structures of *E. coli* EF-Tu·EF-Ts  [26], *Thermus thermophilus* EF-Tu·EF-Ts  [30], and mammalian mitochondrial EF-Tu·EF-Ts  [31], there appears to be room to accommodate an additional methyl group at this position. It is interesting that this change is only observed in *Pseudomonas*.

### 3.2. Expression and Purification of *P. aeruginosa* EF-Tu and EF-Ts

**Figure 2:**
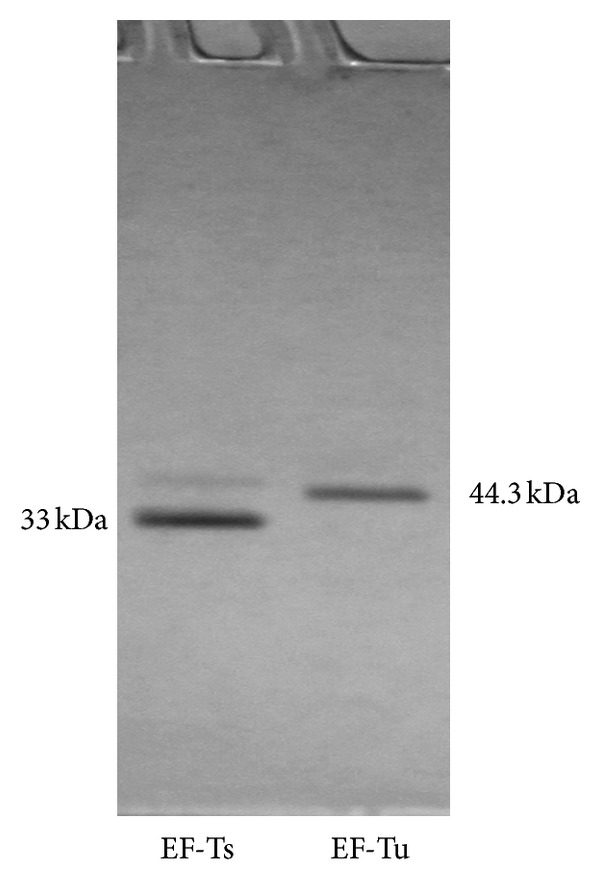
SDS-PAGE analysis of purified *P. aeruginosa* EF-Ts and EF-Tu. Samples (1.0 and 0.5 *μ*g, resp.) of the *P. aeruginosa* EF-Ts and EF-Tu preparations were analyzed on a 4–20% (w/v) SDS-PAGE gel, and the protein bands were visualized by staining with Coomassie blue.

The gene encoding *P. aeruginosa* EF-Ts was PCR amplified and inserted into pET-28b(+) (Novagen). This allowed the protein to be expressed fused to an N-terminal peptide containing a 6-histidine tag for ease of purification. The gene encoding *P. aeruginosa* EF-Tu was PCR amplified and inserted into pET101/D-TOPO (Invitrogen) fused to a C-terminal sequence containing a 6-histidine tag. The DNA sequence of both constructs was confirmed by DNA sequencing, and the plasmids were transformed into Rosetta 2(DE3) (Novagen). Both proteins were expressed in a soluble form, with *P. aeruginosa* EF-Ts comprising approximately 30–40% of the total protein and *P. aeruginosa* EF-Tu appearing to have similar expression as the native *E. coli* EF-Tu (approximately 10% of total protein) but shifted 1 kDa due to the additional amino acid residues in the fusion peptide. The proteins were purified using Ni-NTA resin technology. EF-Tu was purified to approximately 98% homogeneity and EF-Ts to approximately 95% homogeneity (Figure 2). A contaminating protein in the EF-Ts preparation shown in Figure 2 was observed just above the position of *P. aeruginosa* EF-Tu. The masses of *E. coli* and *P. aeruginosa* (plus the fusion peptide) EF-Tu are 43311 kDa and 44267 kDa, respectively, indicating that the contaminating protein was not *E. coli* EF-Tu. Since recombinant EF-Ts, in particular mitochondrial EF-Ts  [32], can bind tightly to the endogenous EF-Tu when overexpressed in *E. coli*, copurification may sometimes occur. To test whether residual *E. coli* EF-Tu was present in the EF-Ts preparation, the GDP exchange assay was used to monitor GDP exchange when only EF-Ts was present in the assay. No GDP binding was observed in these assays (data not shown) indicating that the preparations of EF-Ts used here were free of EF-Tu.

### 3.3. Ability to Exchange Guanine Nucleotides

During purification of *E. coli* EF-Tu the nucleotide GDP is copurified bound to EF-Tu  [33]. Previous analysis indicated that unless EF-Tu is subjected to extensive dialysis (3 days) in the absence of Mg^++^, GDP remains bound to the purified EF-Tu  [34]. To ascertain if *P. aeruginosa* EF-Tu was purified bound to GDP, samples of EF-Tu were heat denatured and precipitated and the concentration of released GDP remaining in solution was measured by absorbance spectroscopy  [35]. From these experiments, the ratio of EF-Tu to GDP was calculated to be at a 1 : 1 ratio, indicating that in fact GDP remained bound to *P. aeruginosa* EF-Tu during the purification process (data not shown).

The percentage of EF-Tu capable of exchanging GDP (active EF-Tu) was determined from assays described in Section 3.6 which measured the total EF-Tu·[^3^H]GDP complexes formed. In these assays, saturating amounts of [^3^H]GDP (12 *μ*M) were incubated with EF-Tu on ice for 2 h, and the total labeled EF-Tu·[^3^H]GDP complex was determined using nitrocellulose filter binding, which retained the complex but not free GDP. The maximum amount of EF-Tu·GDP complex observed would be equivalent to the concentration of EF-Tu capable of exchanging GDP. Approximately 50% of EF-Tu was capable of forming an EF-Tu·[^3^H]GDP complex. In the subsequent experiments containing EF-Tu, the results were based on the concentration of active EF-Tu. 

### 3.4. Stimulation of EF-Tu by EF-Ts

**Figure 3:**
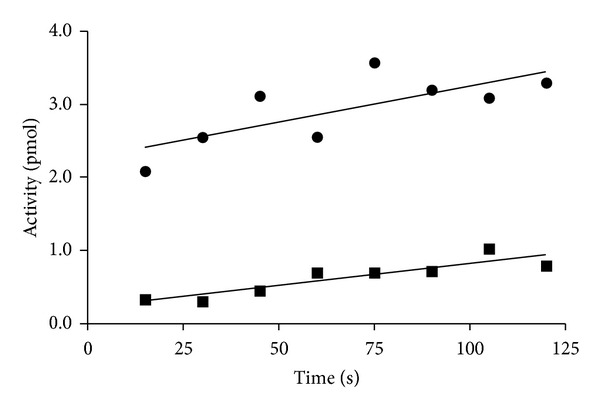
Assays to determine the ability of EF-Ts to stimulate GDPexchange by EF-Tu were as described under Section 2. The concentration of EF-Tu was held at 0.64 *μ*M. EF-Ts concentrations were 0.05 *μ*M (1 : 13 EF-Ts to EF-Tu ratio) when present, and the reaction times for the assays ranged from 15 to 120 s. Activity is the amount of [^3^H]GDP (1500 cpm/pmol) bound by EF-Tu. EF-Tu exchange activity in the presence (●) and absence (■) of EF-Ts.

The catalytic function of EF-Tu is facilitated by EF-Ts which promotes guanine nucleotide exchange. In *E. coli*, EF-Ts promotes the release of GDP from EF-Tu·GDP complex thereby forming an intermediate EF-Tu·Ts complex  [11, 36]. In the absence of GTP and the aminoacylated tRNA, the EF-Tu·Ts complex dissociates to reform the EF-Tu·GDP complex  [34, 37]. To determine the effect of EF-Ts on GDP exchange in the *P. aeruginosa* system, we measured GDP exchange by EF-Tu in the presence and absence of EF-Ts. In reactions containing EF-Ts, the ratio of EF-Ts to EF-Tu was 1 : 13, respectively, which is near the physiological ratio  [38]. The reactions were stopped at 15 s intervals between 15 and 120 s. The average GDP exchanged in the presence of EF-Ts was observed to be 7- to 8-fold greater than that in the absence of EF-Ts (Figure 3). This is similar to the stimulation of EF-Tu by EF-Ts seen in the *E. coli *system  [32].

### 3.5. Examination of Initial Velocity for Interaction of EF-Tu with GDP

**Figure 4:**
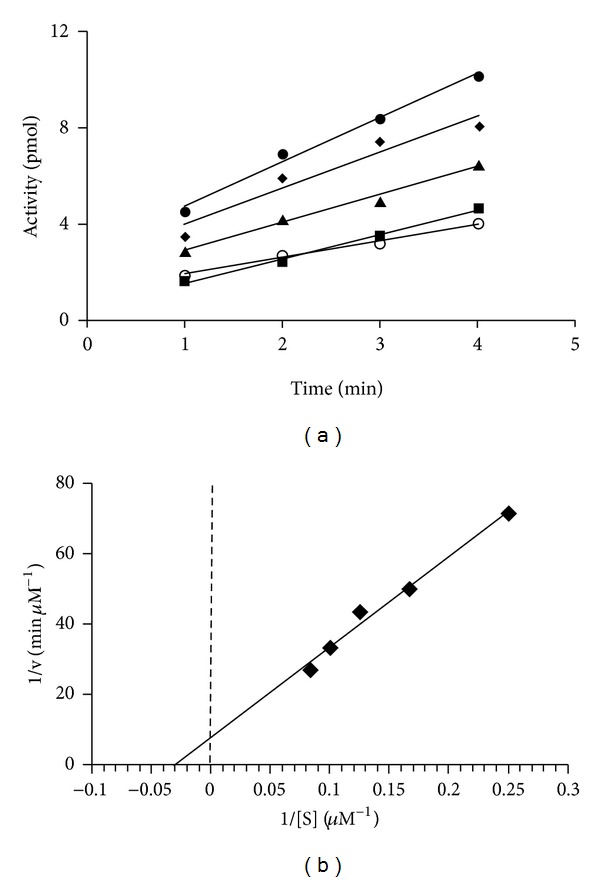
Determination of kinetic parameters for the interaction of EF-Tu with GDP in the absence of EF-Ts. (a) Initial velocities for *P. aeruginosa* EF-Tu in GDP exchange reactions were determined at various concentrations of GDP. The concentration of EF-Tu was held constant at 0.64 *μ*M. The velocities were measured between 1 and 4 min to minimize the chance of measurement of exchange occurring during mixing but before the beginning of the incubation period. The concentrations of GDP were ■, 4 *μ*M; ◯, 6 *μ*M; ▴, 8 *μ*M; *◆*, 10 *μ*M; ●, 12 *μ*M. (b) The data from the initial velocity experiments were used to develop a Lineweaver-Burk plot to determine kinetic parameters for the interaction of *P. aeruginosa* EF-Tu with GDP in the absence of EF-Ts.

**Figure 5:**
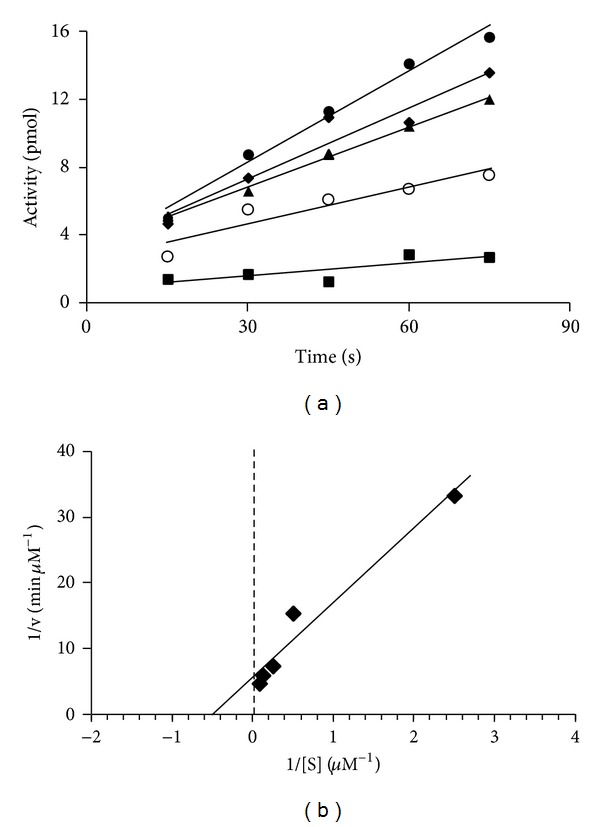
Determination of kinetic parameters for the interaction of EF-Tu with GDP in the presence of EF-Ts. (a) Initial velocities for *P. aeruginosa* EF-Tu in GDP exchange reactions were determined at various concentrations of GDP. The concentration of EF-Tu was held constant at 0.64 *μ*M, and the concentration of EF-Ts was held at 0.01 *μ*M. The velocities were measured between 15 and 75 s. The concentrations of GDP were ■, 0.4 *μ*M; ◯, 2 *μ*M; ▴, 4 *μ*M; *◆*, 8 *μ*M; ●, 12 *μ*M. (b) The data were used to develop a Lineweaver-Burk plot to determine kinetic parameters for the interaction of *P. aeruginosa* EF-Tu with GDP in the presence of EF-Ts.

Historically, the GDP exchange assay has been used to determine the activity of EF-Tu  [20]. From the studies described earlier and elsewhere  [32, 34, 39, 40], it is known that EF-Ts catalyzes the exchange of GDP by EF-Tu. The concentration of GDP in cells is low compared to the concentration of GTP  [38, 41]; thus, it was of interest to determine the kinetic parameters governing the interaction of EF-Tu with GDP both in the presence and absence of EF-Ts. The initial rate of GDP exchange was measured between 1 and 4 min in the absence of EF-Ts (Figure 4(a)). The kinetic parameters KM and Vmax⁡ for the interaction of *P. aeruginosa* EF-Tu with GDP were determined by inserting the initial velocity data into Lineweaver-Burk plots (Figure 4(b)). Alternatively, the kinetic parameters were also determined for the interaction of *P. aeruginosa* EF-Tu with GDP in the presence of EF-Ts. In these assays, the incubation was lowered to times between 15 and 75 s to compensate for the increased rate of the exchange process (Figure 5(a)). Determination of KM and Vmax⁡ values for GDP exchange was carried out at a constant concentration of EF-Tu (0.64 *μ*M) in all assays, and the concentration of EF-Ts was 0.01 *μ*M when present (Figure 5(b)). The KM for the interaction of EF-Tu with GDP in the absence of EF-Ts was 33 *μ*M. We observed a shift of the KM to 2 *μ*M when EF-Ts was added to the reaction. Since this is not a true enzymatic reaction (the reaction does not yield an actual product), we hesitate to address other kinetic parameters such as *k*_cat_ and the specificity constant *k*_cat_/*K*_*M*_ for the interaction. However, previous work has shown that the exchange of GDP by EF-Tu follows first-order kinetics when the concentration of GDP is significantly elevated above the enzyme concentration  [37]. GDP was present in the initial velocity assays at various concentrations to as high as 20-fold and 250-fold above EF-Tu and EF-Ts concentrations, respectively. Therefore, the data which was derived from these experiments yields indirect information on the individual rate constants for the exchange of GDP, and the *k*_cat_ calculated is the observed *k*_cat_. The kcatobs for exchange of GDP by *P. aeruginosa* EF-Tu is 0.003 s^−1^ and 0.005 s^−1^ in the absence and presence of EF-Ts. The specificity constant *k*_cat_^obs^/*K*_*M*_ is 0.1 × 10^−3^ s^−1^ *μ*M^−1^ and 2.5 × 10^−3^ s^−1^ *μ*M^−1^ for EF-Tu in the absence and presence of EF-Ts. The specific activity for the exchange of GDP by EF-Tu was 4.7 × 10^−3^ *μ*mol/min/mg in the absence of EF-Ts. This is almost identical to the specific activity of *E. coli* EF-Tu  [33].

There have been numerous studies analyzing the effect that EF-Ts has on the exchange of GDP by EF-Tu; however, few studies have directly addressed the catalytic parameters of EF-Ts  [37, 39, 42]. In the study by Hwang and Miller [39], *E. coli* EF-Ts catalyzed exchange rates were 5.9–6.2 pmol/min/units of EF-Ts. Assuming the difference between the maximum velocities of EF-Tu in the presence and absence of EF-Ts to be the velocity of EF-Ts, then the turnover number (*k*_cat_^obs^) for EF-Ts can be predicted. The *k*_cat_^obs^ for *P. aeruginosa* EF-Ts was 0.02 s^−1^ resulting in a specific activity of 686 *μ*mol/min/mg.

### 3.6. Determination of the Equilibrium Dissociation Constants of EF-Tu·GDP and the EF-Tu·GTP Complexes

The equilibrium dissociation constants for the EF-Tu·GDP (*K*_GDP_) and EF-Tu·GTP (*K*_GTP_) complexes were calculated from measurements of the extent of dissociation of the complexes in dilute solutions. Both complexes were formed by incubation at 4°C for 2 h in 50 *μ*L reactions (Section 3.3). In these assays, approximately 50% of EF-Tu was observed to form a complex with each form of the nucleotide. To determine dissociation, the 50 *μ*L reactions were diluted by addition of 5 mL of reaction mix (minus enzyme and nucleotide) that had been preincubated to 37°C, 23°C, or 0°C, and incubation was continued at these temperatures for 10, 20, and 30 min, respectively. These were the conditions used to determine the *K*_GDP_ for interaction of *E. coli* EF-Tu with GDP, and we maintained the same conditions in the determination of the *K*_GTP_.

The concentration of complex immediately after dilution was approximately 6.0 × 10^−3^ *μ*M. After incubation, the remaining EF-Tu·GDP complex was determined. Total GDP was calculated as the amount of added [^3^H]GDP plus GDP that had copurified with EF-Tu. Free GDP was the amount of total GDP minus complexes formed, and free EF-Tu was the amount of initial complex minus remaining complex. The equilibrium dissociation constants for the complexes were calculated using the following equation:

(1) $$\begin{array}{c} K_{\text{GD}/\text{TP}}=\frac{\left[\text{EF}-{\text{Tu}}_{\text{free}}\right]\left[\text{GD}/{\text{TP}}_{\text{free}}\right]}{\left[\text{EF}-\text{Tu}\cdot \text{GD}/\text{TP}\right]}. \end{array}$$

We observed KGDP values for EF-Tu that ranged from 30 to 75 nM in the absence of EF-Ts at the temperatures in which the EF-Tu·GDP complex was allowed to dissociate (Table 2). Previous studies to determine the KGDP governing the binding of GDP to EF-Tu in the *E. coli *system included EF-Ts in the assays  [33, 43], and therefore we also carried out assays to determine the KGDP in the presence of EF-Ts. There was no statistical change of the KGDP observed in the presence of EF-Ts (30 to 40 nM). These values are within 3-fold of that seen in the *E. coli* system in which the equilibrium dissociation constant (5 to 10 nM) was determined in the presence of EF-Ts  [33].

For determination of the equilibrium dissociation constants for binding of GTP to EF-Tu the same procedure was used. The KGTP values for EF-Tu were observed to be between 125 and 200 nM in the presence or absence of EF-Ts at the temperatures in which the EF-Tu·GTP complex was allowed to dissociate (Table 3). When the KGTP and KGDP for binding each form of the nucleotide are compared, it becomes apparent that binding of GTP by *P. aeruginosa* EF-Tu is up to 6-fold weaker than binding of GDP. 

### 3.7. Ability of EF-Tu to Form a Ternary Complex and Mediate tRNA Binding to the Ribosome

**Figure 6:**
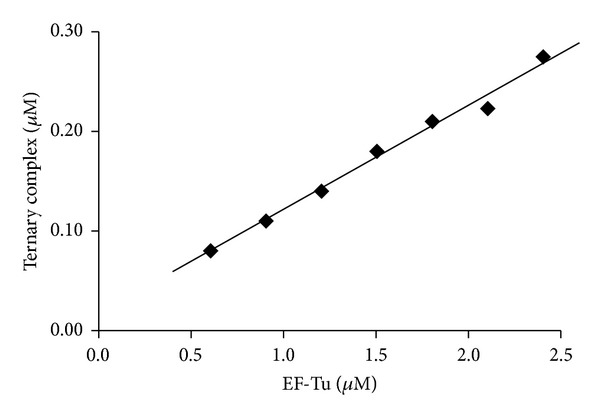
The ability of *P. aeruginosa* EF-Tu to form a ternary complex with aminoacylated tRNA and GTP. Ternary complex formation was analyzed by the ability of EF-Tu to protect [^3^H]Leu-tRNA^Leu^ from digestion by RNase A as described under Section 2. Background activity was subtracted to show only ternary complex formed.

EF-Tu binds aminoacyl-tRNA and GTP to form a ternary complex, and the aminoacylated tRNA is delivered to the ribosome in this form. As a result, for EF-Tu to be functional in protein synthesis, it must be active in ternary complex formation. In *E. coli*, the conformation of EF-Tu in the ternary complex is different than when EF-Tu is bound to either EF-Ts or GDP. The large opening observed between the domains which is seen when EF-Tu is bound to EF-Ts or GDP is closed when EF-Tu is bound to GTP in the ternary complex [44]. To determine the ability of *P. aeruginosa* EF-Tu to form a ternary complex with Leu-tRNA^Leu^ and GTP, the nuclease protection assay was used  [19, 22]. The nuclease protection assay measures the formation of a ternary complex by the ability of EF-Tu to protect the 3′ end of the tRNA with the covalently attached [^3^H]Leu from digestion by RNase A  [45]. [^3^H]Leu-tRNA^Leu^ was used because the concentration of Leu-tRNA in crude *E. coli* tRNA is approximately four times greater than Phe-tRNA, allowing a larger percent of the total tRNA to be charged. Reactions contained enough RNase A to hydrolyze over 90% of the leucine away from tRNALeu. EF-Tu was titrated into the assay, and the amount of Leu-tRNA^Leu^ protected from digestion by RNase A increased in a linear fashion indicating that *P. aeruginosa* EF-Tu was active in ternary complex formation (Figure 6). Up to 20% of the EF-Tu was active in ternary complex formation under the conditions used. This is similar to that observed with *E. coli* EF-Tu  [19].

**Figure 7:**
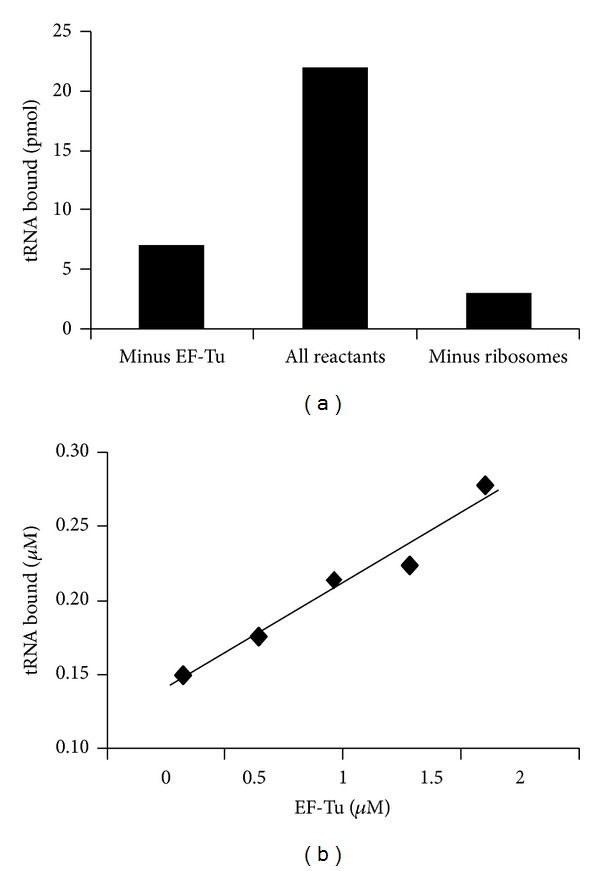
The ability of *P. aeruginosa* EF-Tu to deliver Phe-tRNA^Phe^ to the ribosome in poly(U)-dependent reactions. (a) Requirement of components for binding [^3^H]Phe-tRNA^Phe^ to the ribosome as described under Section 2. Reactions contained 1 *μ*M (50 pmol) ribosome, 0.75 *μ*M [^3^H]Phe-tRNA^Phe^ (37.5 pmol, 25 cpm/pmol), and 3.2 *μ*M EF-Tu. (b) Reactions were as in “A” except that *P. aeruginosa* EF-Tu was varied from 0.32 to 1.6 *μ*M in the reactions. The “tRNA bound” refers to the amount of [^3^H] Phe-tRNA^Phe^ bound to the ribosome. Background activity (minus EF-Tu) was subtracted to show only EF-Tu promoted A-site binding.

Aminoacylated tRNA is bound at the ribosomal A-site in a ternary complex [20]. The amount of aminoacylated tRNAs that can be bound to the A-site of the ribosome in a ternary complex was measured using [^3^H]Phe-tRNA^Phe^ in poly(U)-dependent assays. In the absence of poly(U), a basal level of ternary complex will bind to the ribosome  [20]. In these assays, GTP was replaced with the nonhydrolyzable analog GDPNP to prevent hydrolysis of the nucleotide and dissociation of EF-Tu. As shown in Figure 7(a), EF-Tu promoted binding of the ternary complex to the A-site of approximately 50% of the ribosomes within 15 min. This corresponds to approximately 90% of the ternary complex formed. Removal of EF-Tu or ribosomes in control reactions resulted in approximately 75% and 90% decrease of the signal, respectively. As the concentration of EF-Tu present in the reaction was increased, the binding of the ternary complex to the ribosome increased (Figure 7(b)), and this linear relationship shows the requirement for EF-Tu.

### 3.8. Ability of EF-Tu to Function in Poly(U)-Programed Protein Synthesis

**Figure 8:**
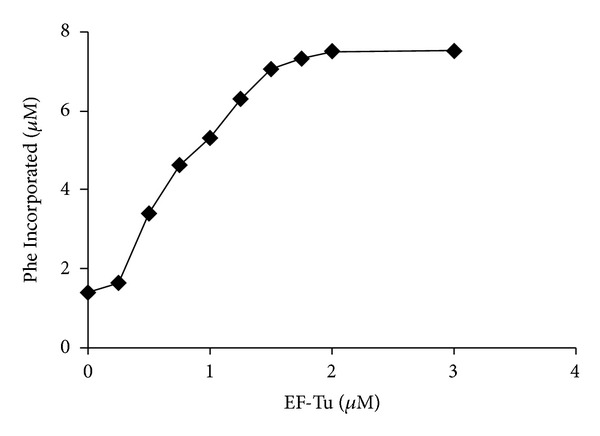
The ability of *P. aeruginosa* EF-Tu to function in poly(U)-directed protein synthesis. The assays were as described under Section 2. Concentrations of EF-Tu were varied between 0.25 and 3.0 *μ*M, and concentrations of ribosomes were held constant at 0.2 *μ*M. “Phe Incorporated” represents the amount of phenylalanine incorporated into peptides during protein synthesis.

An aminoacylation/translation (A/T) system composed of *P. aeruginosa* protein synthesis components has been developed in our laboratory. To determine the ability of EF-Tu to function in protein synthesis, it was titrated into the assay between 0.25 and 3.0 *μ*M (Figure 8). EF-Tu displayed robust activity in polypeptide synthesis. The activity of *P. aeruginosa* EF-Tu is similar to the activity that is seen with *E. coli* EF-Tu in an identical A/T assay with cognate *E. coli *protein synthesis components  [12].

## 4. Discussion

We have described here the cloning and characterization of EF-Tu and EF-Ts from *P. aeruginosa* that will be used in construction of an A/T system for use in screening for antibacterial compounds. EF-Tu has a number of functions in protein synthesis  [25, 26]. EF-Tu has to (1) function in nucleotide (GDP/GTP) binding, (2) interact with EF-Ts to move between the inactive GDP-bound form and the active GTP-bound form, (3) bind aminoacylated tRNA and GTP to form a ternary complex, and (4) be able to deliver the aminoacylated tRNA to the ribosome during protein synthesis. The second elongation factor, EF-Ts, must be able to bind EF-Tu and catalyze the dissociation of GDP and the reassociation of GTP  [11]. We have shown here that *P. aeruginosa* EF-Tu and EF-Ts are active in each of these functions.

We have also shown for the first time that the presence of EF-Ts shifts the *K*_*M*_ for the interaction of EF-Tu with its substrate GDP allowing EF-Tu to function at significantly lower concentrations of GDP. Interestingly, in the absence of EF-Ts, the *K*_*M*_ for the interaction of EF-Tu with GDP was 33 *μ*M. In *E. coli*, the concentration of GDP is approximately 100 *μ*M  [38, 41]. If the concentration of GDP in *P. aeruginosa *cells is similar, then a *K*_*M*_ of 33 *μ*M would assure that in *P. aeruginosa*, EF-Tu would remain bound to GDP after hydrolysis of GTP to GDP in the ternary complex and before EF-Ts acts to recycle the EF-Tu to an active state under physiological conditions. We know that in the absence of GDP, the sulfhydryl group of a cysteine amino acid in the GDP/Mg^++^ binding region interacts with *N*-ethylmaleimide (NEM) resulting in complete inactivation of EF-Tu in *E. coli* [33]. In the presence of GDP, EF-Tu is not inactivated by NEM. This indicates that the sulfhydryl group is exposed to the surroundings and available for contact with an exogenous entity in the absence of GDP, but in the presence of GDP, no contact is possible. This cysteine residue in *E. coli* has been shown to be Cys137 [46]. In the crystal structure of *E. coli* EF-Tu bound to GDP/Mg^++^, Cys137 is located at the surface of the protein and near the nucleotide bind region  [47]. EF-Tu from *P. aeruginosa* has no corresponding cysteine at this position; however, at position 106, there is a cysteine residue (a valine in *E. coli* EF-Tu). A cysteine at this position would be located near Cys137, equally close to the nucleotide binding site and exposed to the surrounding  [47]. Although inactivation of *P. aeruginosa* EF-Tu by NEM has not been shown, this cysteine is a candidate for interaction with NEM. Once EF-Tu hydrolyzes GTP to GDP during the elongation stage of protein synthesis, it remains bound tightly to GDP with a KGDP in the low nanomolar range. However, as we have shown dissociation of GDP from EF-Tu occurs to a certain extent. We suggest that, along with tight binding of EF-Tu to GDP, having a *K*_*M*_ for binding GDP in the range that we have shown may be a mechanism of protecting EF-Tu from inactivation by insuring that it remains bound to GDP.

We have shown that EF-Tu has approximately a sixfold higher affinity for GDP than for GTP. EF-Tu from different organisms binds GDP and GTP with affinities that vary widely. In the *E. coli* system, EF-Tu binds GDP up to 100-fold more tightly than it binds GTP [11]. *T. thermophilus* EF-Tu has a 10–15-fold higher affinity for GDP than for GTP [48]. Alternatively, *Staphylococcus aureus* EF-Tu interacts with GDP with such a low affinity that it is impossible to monitor the interaction [49], as does mitochondrial EF-Tu [50]. However, in all of these organisms, the relative importance of EF-Ts is to function in recycling of EF-Tu from an inactive GDP bound state to an active GTP bound state.

EF-Tu from *Pseudomonas sp.* is all exceptionally similar, with EF-Tu from *P. aeruginosa* being ~90% identical with all other *Pseudomonas sp.* EF-Tu proteins. The degree of homology with EF-Tu from other organisms is also high (from 60 to 80% identity) with the subtle differences noted earlier. However, the degree of homology of *P. aeruginosa* EF-Ts with EF-Ts from all other organisms is much lower (30–50%). In particular, the N-terminal and, to a lesser degree, the C-terminal subdomains of the core region of EF-Ts are quite diverse. Since these are the two regions that interact directly with EF-Tu, it would be of interest in future analysis to evaluate interactions of *P. aeruginosa* EF-Ts with EF-Tu from other organisms.

## Figures and Tables

**Table 1:** Percent identity between *P. aeruginosa* EF-Tu and EF-Ts and the corresponding factors from other organisms.

Species	Evolutionary group	EF-Tu	EF-Ts
*Rickettsia rickettsii *	Proteobacteria; Alphaproteobacteria	77	38
*Neisseria gonorrhoeae *	Proteobacteria; Betaproteobacteria	79	43
*Bdellovibrio bacteriovorus *	Proteobacteria; Deltaproteobacteria	75	40
*Helicobacter pylori *	Proteobacteria; Epsilonproteobacteria	74	32
*Campylobacter jejuni *	Proteobacteria; Epsilonproteobacteria	72	31
*Escherichia coli *	Proteobacteria; Gammaproteobacteria	84	55
*Haemophilus influenzae *	Proteobacteria; Gammaproteobacteria	82	49
*Chlamydia trachomatis *	Chlamydiales/Verrucomicrobia group	67	29
*Corynebacterium jeikeium *	Actinobacteria	68	35
*Mycobacterium tuberculosis *	Actinobacteria	72	31
*Staphylococcus aureus *	Firmicute; Bacilli; Bacillales	73	45
*Streptococcus pneumoniae *	Firmicute; Bacilli; Lactobacillales	68	35
*Clostridium difficile *	Firmicutes; Clostridia	73	45
*Ho* *mo* *sapiens* ^1^	Eukaryota; Opisthokonta; Metazoa	26	12
*Saccharomyces cerevisiae *	Eukaryota; Opisthokonta; Fungi	26	14
*Homo sapiens* mitochondrial	Eukaryota; Opisthokonta; Metazoa	37	19

^1^The protein corresponding to *P. aeruginosa* EF-Tu in eukaryotic cells is EF-1*α*, and the protein corresponding to EF-Ts that was used in the sequence analysis is EF-1*β*.

**Table 2:** Equilibrium dissociation constants for the binding of GDP to *P. aeruginosa* EF-Tu.

EF-Tu_free_^1^ (×10^−3^ *μ*M)	GDP_free_ (×10^−1^ *μ*M)	EF-Tu · GDP (×10^−3^ *μ*M)	EF-Ts (×10^−3^ *μ*M)	Temperature (°C)	*K*_GDP_ (nM)
1.3	1.2	4.5	0.5	37	34
1.2	1.2	4.6	0.5	23	30
1.5	1.2	4.2	0.5	0	43
1.5	1.2	2.5	0	37	72
1.0	1.2	3.2	0	23	38
1.3	1.2	2.7	0	0	58

^1^EF-Tu_free_ and GDP_free_ as described under Section 3.6.

**Table 3:** Equilibrium dissociation constants for the binding of GTP to *P. aeruginosa* EF-Tu.

EF-Tu_free_ (×10^−3^ *μ*M)	GTP_free_ (×10^−1^ *μ*M)	EF-Tu · GTP (×10^−3^ *μ*M)	EF-Ts (×10^−3^ *μ*M)	Temperature (°C)	*K*_GTP_ (nM)
4.2	1.2	2.6	0.5	37	190
3.5	1.2	3.3	0.5	23	125
3.9	1.2	2.9	0.5	0	156
4.1	1.2	2.7	0	37	175
3.5	1.2	3.3	0	23	123
3.8	1.2	2.9	0	0	150
